# Sacral neuromodulation therapy for urinary and defecatory disorders: experience in a Latin American public hospital

**DOI:** 10.61622/rbgo/2024AO11

**Published:** 2024-03-15

**Authors:** Marcelo Mass-Lindenbaum, Diego Arévalo-Vega, Isidora Aleuanlli, Fernanda Santis-Moya, Andrea Maluenda, Eitan Dines, Miriam Cohen-Vaizer, Álvaro Saavedra, Trinidad Raby, Bernardita Blumel, Rodrigo Cuevas, Simone Pohlhammer, Gabriela Alarcon, Marco Arellano Albornoz, Javier Pizarro-Berdichevsky

**Affiliations:** 1 Centro de innovación en Piso Pélvico Hospital Dr. Sótero del Río Santiago Chile Centro de innovación en Piso Pélvico, Hospital Dr. Sótero del Río, Santiago, Chile.; 2 Corporación de Innovación en Piso Pélvico Santiago Chile Corporación de Innovación en Piso Pélvico, Santiago, Chile.; 3 Universidad de Los Andes Facultad de Medicina Santiago Chile Facultad de Medicina, Universidad de Los Andes, Santiago, Chile.; 4 Hospital Las Higueras Talcahuano Chile Hospital Las Higueras, Talcahuano, Chile.; 5 Pontificia Universidad Católica de Chile Departamento de Ginecología Facultad de Medicina Santiago Chile Departamento de Ginecología, Facultad de Medicina, Pontificia Universidad Católica de Chile, Santiago, Chile.; 6 Hospital San Juan de Dios de Los Andes Santiago Chile Hospital San Juan de Dios de Los Andes, Santiago, Chile.; 7 Universidad de Chile Facultad de Medicina Santiago Chile Facultad de Medicina, Universidad de Chile, Santiago, Chile.; 8 Universidad del Desarrollo Facultad de Medicina Clínica Alemana Santiago Chile Facultad de Medicina Clínica Alemana, Universidad del Desarrollo, Santiago, Chile.; 9 Clínica Santa María Santiago Chile Clínica Santa María, Santiago, Chile.; 10 Clinica BUPA Santiago Santiago Chile Clinica BUPA Santiago, Santiago, Chile.; 11 Red de Clínicas y Centros Médicos RedSalud Santiago Chile Red de Clínicas y Centros Médicos RedSalud, Santiago, Chile.; 12 Clínica Puerto Varas Puerto Varas Chile Clínica Puerto Varas, Puerto Varas, Chile.

**Keywords:** Sacral neuromodulation, Urinary bladder, overactive, Non-obstructive urinary retention, Fecal incontinence, Incontinence, Electric stimulation therapy

## Abstract

**Objective:**

To show the experience of a Latin American public hospital, with SNM in the management of either OAB, NOUR or FI, reporting feasibility, short to medium-term success rates, and complications.

**Methods:**

A retrospective cohort was conducted using data collected prospectively from patients with urogynecological conditions and referred from colorectal surgery and urology services between 2015 and 2022.

**Results:**

Advanced or basic trial phases were performed on 35 patients, 33 (94%) of which were successful and opted to move on Implantable Pulse Generator (GG) implantation. The average follow-up time after definitive implantation was 82 months (SD 59). Of the 33 patients undergoing, 27 (81%)reported an improvement of 50% or more in their symptoms at last follow-up. Moreover, 30 patients (90%) with a definitive implant reported subjective improvement, with an average PGI-I "much better" and 9 of them reporting to be "excellent" on PGI-I.

**Conclusion:**

SNM is a feasible and effective treatment for pelvic floor dysfunction. Its implementation requires highly trained groups and innovative leadership. At a nation-wide level, greater diffusion of this therapy among professionals is needed to achieve timely referral of patients who require it.

## Introduction

Sacral neuromodulation consists in stimulating the S3 sacral nerve roots, with a minimally invasive procedure. It has excellent long-term outcomes in overactive bladder (OAB), idiopathic non-obstructive urinary retention (NOUR) and fecal incontinence (FI).^(1,2)^

According to the International Continence Society (ICS), OAB with incontinence is defined as an involuntary leakage of urine, associated with a sudden compelling desire to void.^2^ It affects nearly 50% of women, and the percentage of affected women increases with age.^(3)^

Idiopathic NOUR is a condition in which there is an incapacity to empty the bladder, without obstruction to the urinary flow and without an underlying neurologic condition. It is a uncommon condition, but it can lead to kidney damage and/or recurrent urinary infections.^(1)^

FI is a common and debilitating condition defined as the uncontrolled passage of feces or gas. This pathology has a negative effect on quality of life, leading to social isolation. Its prevalence varies from 7% in the general population and up to 50% in institutionalized patients.^(4,5)^ The diagnosis of OAB is clinical, with symptoms such as urgency, urge-incontinence, and increased daytime-nighttime frequency, in the absence of a urinary infection.^(2)^ According to various clinical guidelines, the treatment pathway for OAB is staged, with the first line consisting in behavioral changes, the second line consisting in pharmacological therapy with antimuscarinics or Beta-3 agonists, and posterior tibial nerve neuromodulation (PTNS). The third line of management consists in either sacral neuromodulation (SNM) or botulinum toxin injection into the detrusor muscle.^(6)^

In the case of NOUR, the diagnosis must be confirmed with a urodynamic study that rules out obstruction. The suggested first-line treatment is clean intermittent catheterization; however, some patients are unable to perform it or it is not the best option for those patients. In these cases, SNM should be considered as a therapeutic option.^(7)^ In the case of FI, the diagnosis is clinical, and the American Society of Colon and Rectal Surgeons (ASCRS) states that after failing biofeedback, the next option is neuromodulation in the absence of recent sphincter damage.^(8)^ SNM involves the implantation of a pulse generator or IPG (Implantable Pulse Generator) connected to a quadripolar electrode to stimulate the sacral roots. The available IPG battery in our country (InterStim II™) lasts 4-6 years.

In the literature, it is described that after SNM implantation for the management of refractory OAB, 60-90% of patients report symptomatic improvement and 30-50% report complete symptomatic relief.^(9)^ This treatment has been approved by the Food and drugs administration (FDA) for NOUR, with success rates between 70-80% and has also been approved by the FDA for FI, achieving continence rates of over 90%. Therefore, it is an excellent therapy for patients with refractory dual incontinence, meaning patients with both FI and OAB, as it is the only therapeutic option capable of treating both conditions with a single procedure.^(10)^

This retrospective study aims to share our experience with SNM in the management of refractory OAB syndrome or NOUR and/or FI, reporting feasibility, short to medium-term success rates, and complications.

## Methods

We present a retrospective cohort using data collected prospectively from patients with urogynecological conditions and referred from colorectal surgery and urology services from the Sótero del Río Hospital, in Santiago, Chile, between 2015 and 2022. This study was authorized by the Research Ethics Committee.

We included female patients with refractory OAB, NOUR and/or FI, who, according to the ICS, the International Urogynecology Association (IUGA) and the ASCRS require SNM as a treatment for their pathologies.^(10)^ All NOUR patients had a urodynamic study prior to offering SNM.

Candidates were informed of the risks and benefits of SNM and signed an informed consent form. The population belonged to the public health system; therefore, the cost was absorbed by the hospital budget, with no costs for the patients. All surgeries were performed by sub-specialists in pelvic floor reconstructive surgery trained in SNM at Cleveland Clinic, Ohio.

The procedure is performed in two phases. The first phase can be either a basic trial (Peripheral nerve evaluation) or an advanced trial ("Stage I"). The second phase refers to the long-term implantation of the device, full implant in case of a successful peripheral nerve evaluation (PNE) or implant of the generator (or "Stage II") in case of a successful stage I, details will be explained in the following paragraphs. The trial phase helps to define which patient will benefit from this therapy.

Voiding (with post-void residue for urinary retention) and/or bowel (in the case of FI) diaries were used at baseline and compared with diaries after basic or advanced trials, to objectively measure improvement.

The accepted definition^(10)^ of a successful trial is one in which there is a 50% or more symptomatic improvement compared to the baseline prior to the basic or advanced trial phase.

In general, since 2019, as recommended,^(10)^ we prefer to do, as a first approach, a basic trial in all our cases. PNE is performed with temporary monopolar electrodes that are implanted in a procedure room, with or without fluoroscopic guidance (in our center we never use fluoroscopy), unilaterally or bilaterally, under local anesthesia without sedation. These electrodes are connected to a temporary pulse generator for a 7-14 days period.

**Figure 1:**
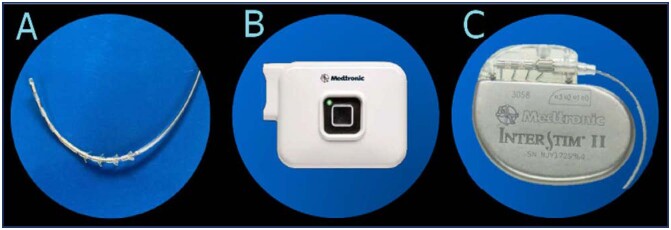
A: Spiculated Quadripolar electrode. B: Temporary Pulse Generator. C: Implantable Pulse Generator (Interstim II)

If the PNE is successful, then a single surgical procedure is performed where the definitive spiculated quadripolar electrode and generator are implanted (also known as "full implant"). If the PNE is not successful, an advanced trial should be performed. This way, a successful PNE reduces the need of multiple operating room procedures to a single procedure, which makes it easier and accessible for patients and could be more cost-effective. The advanced trial (or "Stage I") consists in implanting the electrode, guided by fluoroscopy, under local anesthesia and sedation. A spiculated quadripolar electrode (Figure 1) is connected to a temporary pulse generator for a period of 2-3 weeks.

**Figure 2:**
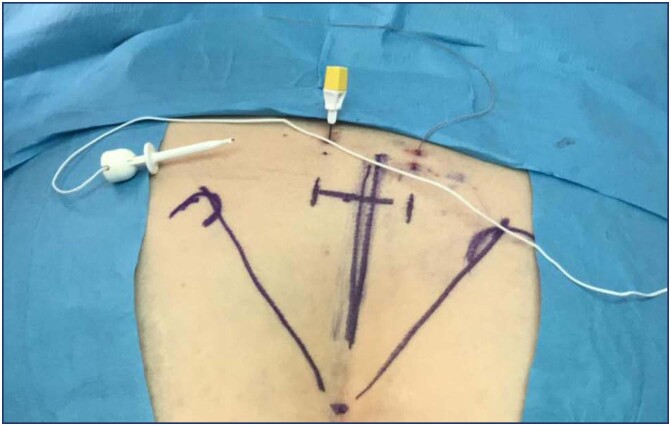
Peripheral nerve evaluation anatomic landmarks. Insulated needle on the left and electrode already delivered on the right side

If the advanced trial is successful, the definitive generator implantation is planned, also known as "Stage II". If it fails the neuroelectrode is removed and an alternative therapy should be pursued. Both basic and advanced trial techniques aim for the electric impulse to achieve S3 motor response with flexion of the first toe and contraction of the anal bellows and perineal sensitive response at the lowest possible voltage (desirable < 2 volts in 4 electrodes).^(11)^ The use of PNE started in 2019 once it was available in our country. The kit contains everything needed to perform the test, except lidocaine and antiseptic solution. Every case was performed in a procedure room, bilaterally, under local anesthesia without fluoroscopy and without sedation. The technique is based on pelvic bone anatomy landmarks: the coccyx is identified by palpation, marking with a surgical pen at 9, 10, and 11 cm cephalad from the coccyx at the midline, then 2 cm laterally from the marks already described, which should roughly correspond to the S3 foramina (Figure 2).

**Figure 3:**
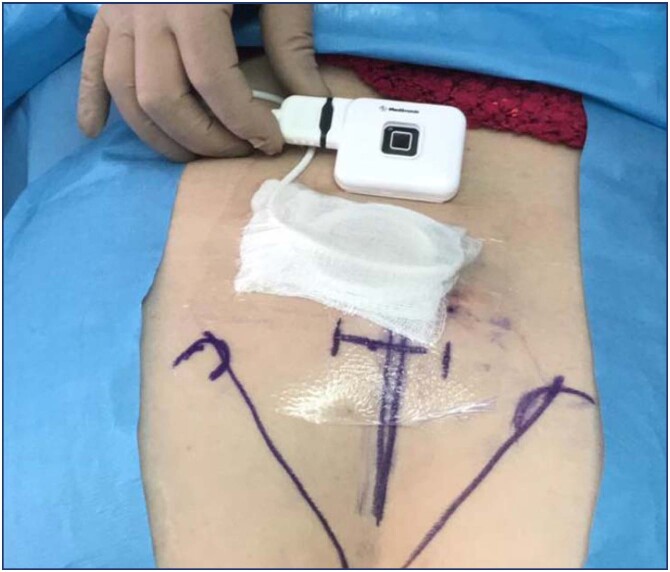
Peripheral nerve evaluation electrodes connected to the external stimulator

Then, with local anesthesia, the electrically insulated needle is introduced, and the desired motor-sensory response is looked for. When the best motor and sensory response is obtained, the temporary electrodes are inserted through the trocar working channel bilaterally, which are connected to the external stimulator (Figure 3). Bilaterality allows the stimulation side to be changed during the trial.

**Figure 4:**
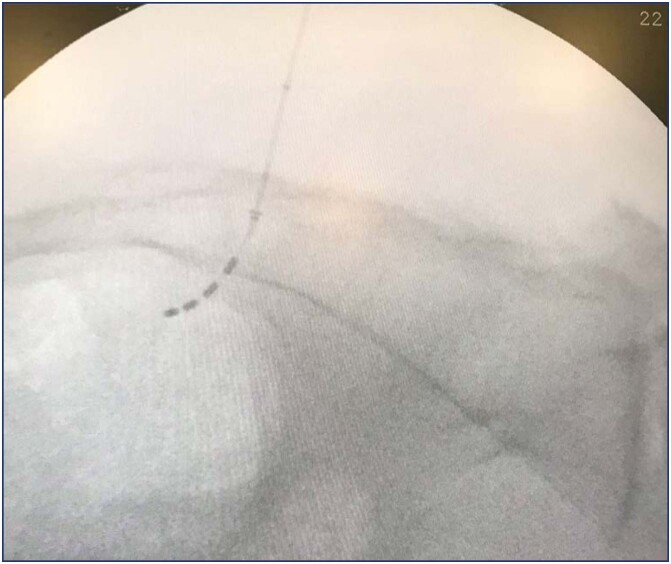
Lateral view of optimal electrode placement, following the sacral root from cephalad to caudal direction

**Figure 5:**
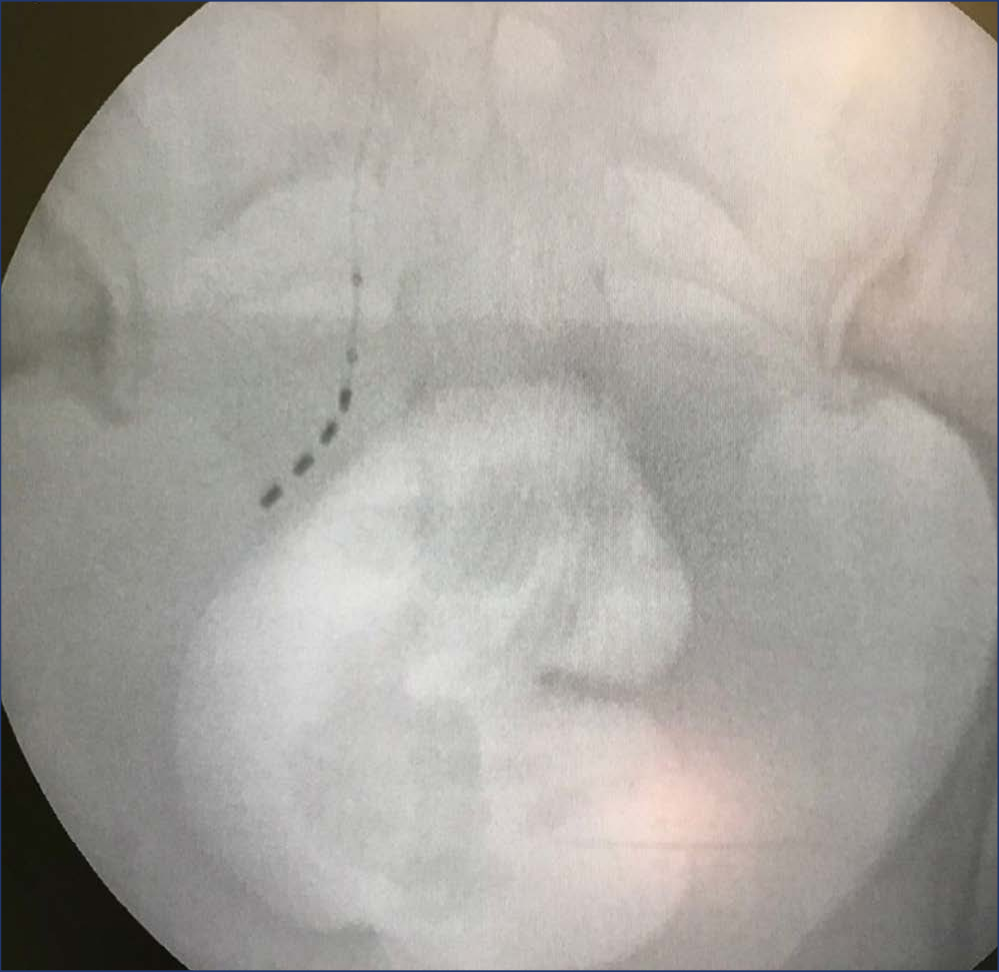
Antero-posterior view of optimal electrode placement, following the sacral root from medial to lateral, at the level of the sacroiliac joint where the S3 foramina is located

For the advanced trial (or "stage I"), fluoroscopy and sedation are used, once the S3 response is achieved, the quadripolar electrode is introduced (Figures 4 and 5), connected to the external generator via an extension cable; if the test is not successful, the patient goes to the operating room to remove the spiculated quadripolar electrode and extension cable.

**Figure 6:**
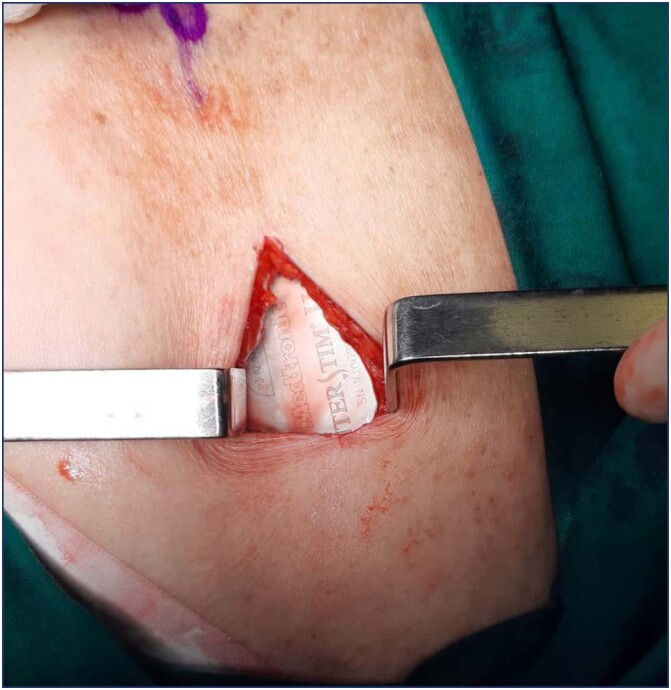
IImplantable Pulse Generator in subcutaneous pocket

After 7 to 21 trial days (depending on whether it was basic or advanced) patients with objective improvement on voiding diaries and/or fecal diaries progress to the final pulse generator implantation, InterStim II™ (Figure 6), in a subcutaneous pocket for advanced trial patients and "full implant" for successful basic trial patients. All patients were followed up at 4-6 weeks after the permanent device was implanted, and according to their subjective and objective response, monthly reprogramming was performed until the best response was obtained. Then, regular check-ups were done at 3 to 6 months periods and then annually.

Regarding the device programming, per manufacturer recommendations, there are 7 different stimulation combinations: 4 basic programs (single negative) and 3 advanced programs (double negative). When a patient had a good response during the trial phase, but later on the symptomatic improvement was lost or did not reach the same as during the trial phase, reprogramming was performed starting with the basic single-negative and then with the advance double-negative programs in the Medtronic programmer. Each basic and advanced program is trialed for at least 1 month between each change to better evaluate symptomatic improvement. If, after evaluating all programs, 50% or more symptom improvement is not achieved, a revision or change of the neuroelectrode is performed. A descriptive analysis of demographic variables was performed. The success rate of SNM was also described based on an improvement in symptoms of greater than or equal to 50%. Complications associated with the surgical procedure and follow-up times are described, along with the assessment of subjective outcomes with the Patient Global Index Improvement (PGI-I) survey, validated for pelvic floor pathologies.^(12)^

## Results

Advanced or basic trial phases were performed on 35 patients, 33 (94%) of which were successful and advanced to IPG implantation. There were no intraoperative complications associated with the surgical procedure, both in the trial phase and in the definitive implantation. The average operative times were 72 minutes for stage I, 26 minutes for stage 2 and 45 minutes for PNE. Demographic characteristics are described in table 1.

**Table 1:** Demographic characteristics

Demographic characteristics	mean/SD)/ n(%)
Total patients	35
Age	63.2 / 10.7
BMI	30.1 / 7.5
Menopause	26(74.2)
Tobacco use	11(31.4)
Parity	3/1
Stress urinary incontinence	9 (25.7)
Pelvic organ prolapse	5(14.3)
Depression	7(20)
Hypertension	17(48.6)
Diabetes Mellitus	5(14.3)

**Figure 7:**
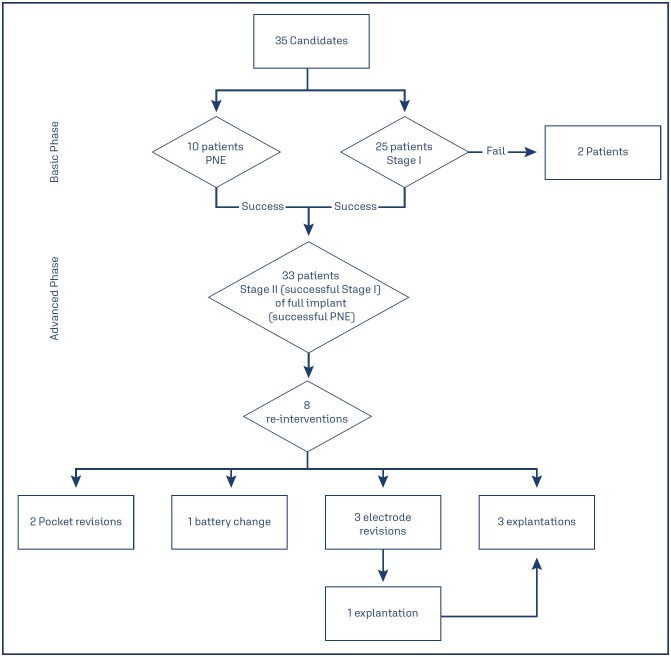
Flow of patients

Most of the patients (33/35) had OAB, of whom 10 also had fecal incontinence (FI) associated, or dual incontinence. Only two patient had NOUR. All OAB patients, prior to SNM, tried second-line treatment with antimuscarinic and/or transcutaneous PTNS (t-PTNS) and three of them advanced to third-line treatment with intradetrusor botulinum toxin injections. All OAB patients were OAB-"wet", meaning they all had urge-incontinence associated to their symptoms. None of the patients had neurogenic OAB. The average follow-up time after the definitive implantation was 82 months (SD 59). Of the 33 fully implanted patients, 27 (81%) reported an objective improvement of 50% or more of their symptoms at last follow up. Eight of the 33 patients were re-intervened. Of the Eight re-intervened patients, three underwent electrode revision, one of whom lost therapeutic response due to electrode migration after trauma to the gluteal region. Another patient had suboptimal response despite reprogramming, and another patient who, despite having successful response presented painful flexion of the 1st metatarsal joint. These three patients achieved satisfactory response without adverse effects after revision, one patient, 2 years after the electrode revision, had a non-infectious inflammatory response to the IPG and had to have the IPG removed. Two patients underwent battery pocket revision due to persistent pain with pain improvement after revision. One patient had the battery changed at the end of its lifespan after 6 years post-implant, recovering excellent clinical response. Three patients had the neuromodulator explanted, one due to painful stimulation, another due to a non-infectious pocket inflammatory response, as detailed above, and another due to lack of response despite the use of basic and advanced programs, requesting removal. The flow of patients is summarized in figure 7.

**Figure 8:**
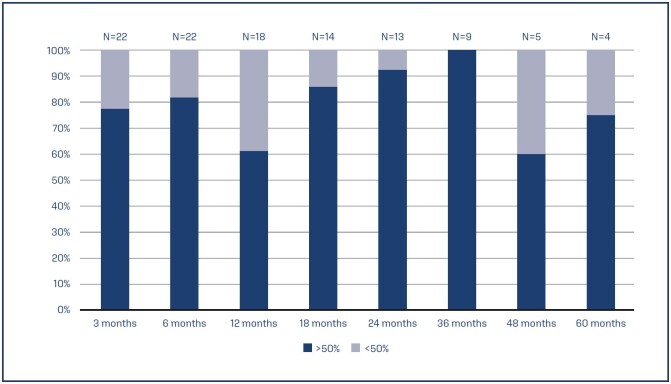
5 years follow up of symptomatic improvement, defined as more than 50% compared to baseline

Of the 10 patients with FI, 9 reported great objective and subjective improvement incontinence episodes during follow-up. After the last follow-up visit, 30 patients (90%) with a definitive implant reported subjective improvement, with an average PGI-I "much better" and 9 of them reporting to be "excellent" on PGI-I. In regards of continence, in their last follow-up visit, 4 (11.4%) of the patients reported being 100% continent. During follow-up, 29 patients required reprogramming, with an average of 2 times per patient. In 10 patients, advanced programs were used. 26 of the 29 patients had improvement of more than 50% in their symptoms after reprogramming, without the need for electrode revision. Medium-term results show that at 24 and 36 months more than 90% of the patients reported improvement of more than 50% of symptoms (Figure 8).

## Discussion

SNM has been a widely used treatment for pelvic floor dysfunctions for more than 25 years since its FDA approval and over 300,000 patients have been implanted worldwide. Its implementation has changed the lives of many patients, allowing them to resume their activities and reintegrate into society. Our results are comparable to those reported in the international literature, both in the success rates of the trial phase and long-term results. Our experience is one of the first to be reported in Latin America despite the 25 years of existence of SNM. Some of the difficulties in implementing this therapy in our region are due to the high costs and lack of insurance coverage. For our institution, the cost of supplies exceeds CLP$9,000,000 (US$12,000) per implanted patient. However, for OAB patients the alternative third line therapy to SNM is botulinum toxin, using the dose (200U) published in the Rosetta^(13)^ study, after 5 years with reinjections every 6-8 months, this could end up being even more expensive, even with 100 U injections.

Currently, in our country, SNM has been recently incorporated into the public health system, including 5 FONASA (public health insurance) codifications according to the procedure, only available in public hospitals. However, there is still no coverage for the implantable pulse generator - being the most expensive part of the therapy - generating a greater challenge in health equity.^(14)^

So far, all implants have been funded with the hospital budget allocated by the Health Ministry. This has been possible based on the visionary leadership of our hospital, its authorities, and the clinical team, understanding the social responsibility of the institution for a low-income and high social risk population. This codification is not available for the private health system in Chile, therefore patients with private health providers do not have any type of financial coverage for this therapy at the time.

Given the high cost of the therapy, the volume of patients is lower therefore affecting the learning curve of the surgical technique which impacts the long-term outcome.^(15)^

It seems that by now, our country should have a national reference center to train more specialists until we have better economic and territorial coverage of this therapy. During our experience, a third member of our service, trained in this technique in Canada, joined our team, and recently, a fellow was trained in SNM in our institution, but is currently working in another hospital. According to Medtronic data, the sole supplier for our country, there are 2 other surgeons trained in Chile, only one of them in the public system. This means that most of the surgeons trained in SNM are part of our institution. For this reason, as previously mentioned, we believe that our hospital should be the national referral center for the management of patients who require this therapy, until there is capacity to train more surgeons, which we hope can be solved in the medium-long term. Implementing this therapy involves a continuous commitment to patients, keeping them under frequent follow-up visits to look for the need for reprogramming, which as our results show, most will need. In our case, the reprogramming allowed us to regain a satisfactory clinical response, thus avoiding new surgeries to check the electrodes, as shown in the study by Pizarro-Berdichevsky et al.,^(11)^ a suboptimal motor response in the operating room was associated with a higher rate of electrode revision and the authors suggest that this could be due to lower options to reprogram the system and in that study, the authors found that optimal response in all 4 electrodes was associated with lower rates of electrode revisions; if this is true it could reflect that our surgical technique has been refined and with it, our reprogramming has been successful. Our study has several strengths. One of them is that to date, there are no Latin American studies reporting the success of SNM in this region, as a standard of treatment and not as case reports. Another strength is that the surgical team that performs SNM is highly trained in world-renowned centers. Our hospital is a high-volume hospital, in fact it is the hospital that serves the biggest population in ChiIe.

We also show that SNM is feasible and effective and we show a model of how public hospitals can arrange treating OAB, NOUR and FI. Regarding the limitations, our study is of retrospective and observational natures, which should be taken into consideration for making clinical decisions. We also note that our number of patients is very small, but is currently increasing, thanks to our program which treats more and more patients. It is noteworthy that to date, we have not required any patient to advance to a fourth line of treatment with a bladder augmentation which is a high morbidity surgery. Therefore, it is important that this therapy be covered by public and private systems, providing health equity. Similarly, in patients with FI, studies in the Spanish health system^(15)^ show the efficacy with Quality adjusted life years (QALYs), and symptom-free years, and conclude that SNM is an effective and cost-effective population measure. At a nation-wide level, greater diffusion of this therapy among professionals is needed to achieve timely referral of patients who require it.

## Conclusion

Sacral neuromodulation is a feasible and effective treatment for the treatment of pelvic floor dysfunctions (90%) with a definitive implant reported subjective improvement, with an average PGI-I "much better" Its implementation requires highly trained groups and innovative leadership.
